# Nondestructive Detection of Egg Freshness Based on Infrared Thermal Imaging

**DOI:** 10.3390/s23125530

**Published:** 2023-06-13

**Authors:** Jingwei Zhang, Wei Lu, Xingliang Jian, Qingying Hu, Dejian Dai

**Affiliations:** 1 School of Electrical and Electronic Engineering, Anhui Science and Technology University, Bengbu 233000, China; 2 College of Artificial Intelligence, Nanjing Agricultural University, Nanjing 210031, China; jianxingliang@njau.edu.cn (X.J.); 2022119017@stu.njau.edu.cn (Q.H.); djdai@njau.edu.cn (D.D.)

**Keywords:** egg freshness, infrared thermal imaging technology, finite element analysis of heat transfer

## Abstract

In this paper, we proposed a nondestructive detection method for egg freshness based on infrared thermal imaging technology. We studied the relationship between egg thermal infrared images (different shell colors and cleanliness levels) and egg freshness under heating conditions. Firstly, we established a finite element model of egg heat conduction to study the optimal heat excitation temperature and time. The relationship between the thermal infrared images of eggs after thermal excitation and egg freshness was further studied. Eight values of the center coordinates and radius of the egg circular edge as well as the long axis, short axis, and eccentric angle of the egg air cell were used as the characteristic parameters for egg freshness detection. After that, four egg freshness detection models, including decision tree, naive Bayes, k-nearest neighbors, and random forest, were constructed, with detection accuracies of 81.82%, 86.03%, 87.16%, and 92.32%, respectively. Finally, we introduced SegNet neural network image segmentation technology to segment the egg thermal infrared images. The SVM egg freshness detection model was established based on the eigenvalues extracted after segmentation. The test results showed that the accuracy of SegNet image segmentation was 98.87%, and the accuracy of egg freshness detection was 94.52%. The results also showed that infrared thermography combined with deep learning algorithms could detect egg freshness with an accuracy of over 94%, providing a new method and technical basis for online detection of egg freshness on industrial assembly lines.

## 1. Introduction

Eggs are rich in proteins, vitamins, minerals, fats, and other nutrients required by the human body and thus have an important place in the dietary pyramid. The quality of eggs affects farm grading and sales, egg processing, product quality, and consumer purchase desire [1]. Under the influence of storage time, the storage environment, and vibrations during transportation, minerals inside the egg are exchanged between the albumen and yolk through the yolk membrane. Water and carbon dioxide are exchanged with the outside world through the shell pores, resulting in liquefaction of the albumen, an increase in pH, flattening of the yolk, growth of the air cell height, and thinning of the shell, all of which cause loss of nutrients and deterioration of egg quality [2]. Therefore, nondestructive testing of egg freshness, the most important indicator of egg quality, is particularly important. Currently, egg freshness is measured by Haugh unit (HU), yolk coefficient (YC), and air cell height (ACH) [3].

Traditional egg freshness testing methods rely on human observation and destructive testing, which are slow, require specialized personnel, and do not meet the needs of modern testing. Because eggs produce ammonia oxides, alkanes, alcohols, and other gases during storage [4], Dutta et al. built an electronic nose system to measure these gases using four supervised classifiers of egg freshness, with an accuracy of 95% [5]. Yimenu, Kim, and Kim determined the proportion of different gas concentrations on the egg surface according to gas chromatography and verified differences in the volatile characteristics of egg samples at different storage times by principal component analysis (PCA) and discriminant factor analysis (DFA); this study demonstrated the feasibility of using a fast gas chromatography electronic nose for predicting egg freshness [6]. However, the electronic nose detection method is only suitable for sampling detection and is vulnerable to the influence of external gases in the environment; furthermore, the detection speed is slow and unsuitable for pipeline detection. Finally, using conductivity technology [7] and dielectric spectroscopy [8,9] methods to determine egg freshness is time-consuming and not widely applicable.

To achieve rapid detection of egg freshness, spectral technology has been applied to the field. Aboonajmi et al. established a radial basis function network model by collecting the spectral data of eggs in the wavelength range of 30–1100 nm based on visible near-infrared spectroscopy technology, which had good prediction accuracy for HU and ACH [10]. Cavanna et al. combined ion mobility spectrometry (IMS) with gas chromatography (GC) to classify eggs with different freshness levels, and 97% of the samples were correctly classified [11]. Dai et al. studied scattered hyperspectral, reflectance hyperspectral, transmission hyperspectral, and hybrid hyperspectral imaging for detection of egg freshness and found that the detection accuracy of scattered hyperspectral imaging was the highest, and the detection accuracy was inversely proportional to the magnitude of the incident angle [12]. Although spectroscopic techniques can detect egg freshness nondestructively, the data are large, variable, limited in detection range, and highly susceptible to eggshell thickness and ambient light factors.

Machine vision has been the most widely used method for detecting egg freshness in recent years. Sun et al. collected egg size and weight information based on machine vision and a dynamic weighing system [13]. They established an HU prediction model using multiple linear regression (MLR), with a correlation coefficient (R) value of 0.8653. Similarly, a linear relationship between egg density and freshness was determined using a machine vision system to estimate egg volume and a weighing system to measure weight [14]. Tan et al. used nuclear magnetic images to establish the correlation between the centroid rate and HU based on the change in egg moisture with storage time [15]. Although the use of machine vision technology is relatively mature in all fields [16,17,18], this method is susceptible to the effects of shell thickness and surface dark spots when testing egg freshness.

With the development of smart agriculture, machine learning and deep learning have been widely used in the field of agricultural product inspection [19,20,21,22]. Meanwhile, nondestructive thermal infrared imaging techniques are widely used for quality inspection of agricultural products [23,24,25,26,27,28], and they may also be applied to egg freshness inspection.

In this study, we used eggs as the research object. To perform egg freshness detection on industrial assembly lines, we proposed an egg freshness detection scheme based on infrared thermal imaging and a SegNet neural network. The main contributions of this study are as follows: (1) simulation analysis was conducted on the thermal response characteristics of eggs. The results of this simulation yielded feature parameters for egg freshness detection and determined the relationship between the height of the egg air cell and the feature information within egg thermal infrared images. (2) Machine learning and a SegNet neural network were used to collect egg feature information, and five egg freshness detection models were established to effectively distinguish the freshness of different eggs. Compared to conventional machine vision for egg freshness detection, this method provided more information on the egg characteristics and was suitable for detecting eggs with different shell colors and cleanliness levels. The method proposed in this paper can provide a reference for the development of equipment to detect egg freshness on factory lines.

## 2. Materials and Methods

### 2.1. Thermal Excitation Temperature and Time Optimization Based on Finite Element Analysis of Egg Heat Transfer

#### 2.1.1. Fluid and Heat Transfer Solutions

We used ANSYS Mechanical APDL 19.2 software to solve the heat transfer process near the egg’s blunt end upon hot air impact. To simplify the solving process, the thermal and physical properties of the egg were assumed to be constant with temperature, and the difference between egg albumen and yolk was ignored.

#### 2.1.2. Geometry and Grid Generation in ANSYS

The first step of an ANSYS analysis is to design the system geometry and discretize it into a computational grid of control volumes. The geometry of the egg was obtained by rotating the egg profile equation around the axis of symmetry [29]:

(1) $$\frac{x^{2}}{a^{2}}+\frac{y^{2}}{{\left(b+x\tan\theta \right)}^{2}}=1$$

where *a* is the long axis radius of the egg; *b* is the short axis radius of the egg; *θ* is the egg shape angle; and *x* and *y* are the horizontal and vertical coordinates of points on the curve, respectively. The *a* and *b* values for the egg used in the experiments were 28.435 and 21.89 mm, respectively, and the *θ* value was 10°. The eggshell thickness was set to 0.35 mm.

**Figure 1:**
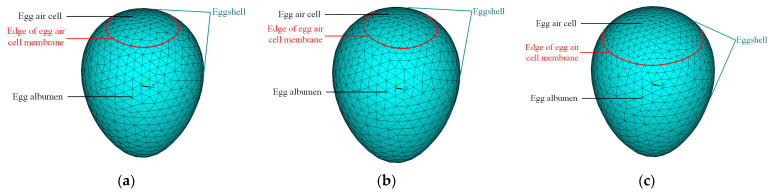
Schematic diagrams of meshing of different egg models. (**a**) H-EGG, (**b**) S-EGG, and (**c**) N-EGG.

To analyze the temperature field of eggs with different freshness levels after instantaneous heating, we established three geometric models in this paper: H-EGG, horizontal air cell and ACH = 3 mm; S-EGG, sloped air cell and ACH = 3 mm; and N-EGG, horizontal air cell and ACH = 9 mm (Figure 1).

#### 2.1.3. Initial and Boundary Conditions

Many scholars have studied the egg heat transfer process. Sabliov et al. used an axisymmetric unsteady finite element heat transfer model to simulate the process of eggshell cooling by low-temperature carbon dioxide, during which the eggs were considered to be composed of isotropic yolk, albumen, air cell, and shell [30]. Denys, Pieters, and Dewettinck subjected eggs filled with viscous liquid to water bath heating and found that the presence of spherical yolks did not affect the overall heat transfer process [31]. Erdogdu et al. used a slit air impingement system to cool hard-boiled eggs and simulated different cooling conditions, finding that the system was more effective in cooling hard-boiled eggs than immersion cooling with water [32].

The relevant conditions for this study were as follows:

We assumed that the egg’s overall initial temperature distribution was constant at 10 °C, and the heat transfer caused by egg surface radiation was ignored.

To ensure the stability of the internal chemical structure of the eggs and to highlight temperature changes, we used hot air at 20, 25, 30, 35, 40, and 45 °C to heat the egg’s blunt end.

The egg contents and the gas chamber were uniformly distributed as a solid treatment with no mutual flow.

The thermal and physical properties used in the model are organized in Table 1 [33].

#### 2.1.4. Post-Processing Analysis

**Figure 2:**
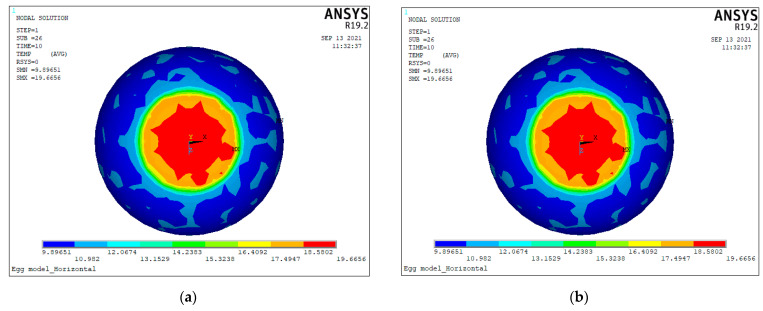

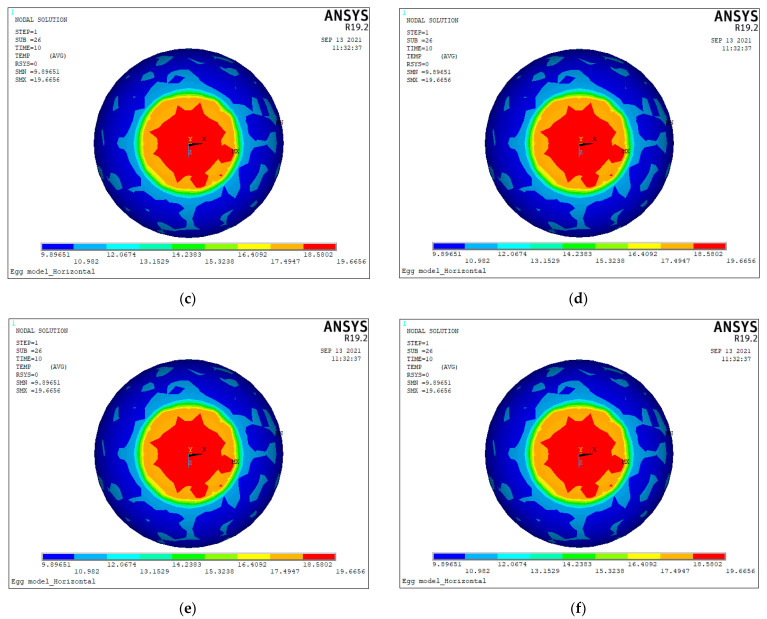
The temperature fields of H-EGG blunt end after applying deferent thermal excitation for 10 s. (**a**) 20 °C, (**b**) 25 °C, (**c**) 30 °C, (**d**) 35 °C, (**e**) 40 °C, and (**f**) 45 °C.

The temperature fields of the blunt end in the H-EGG model heated by thermal excitation at different temperatures for 10 s are shown in Figure 2. Under different thermal excitation temperature conditions, the overall temperature changed in the same pattern, even though the final temperature values of the eggs were different. Because the infrared thermal imager was sensitive, a prominent boundary line was generated in the thermal infrared image once a temperature difference existed between the egg air cell and albumen. After comparing simulated images of thermal excitation at different temperatures and several trials, we performed transient heating of the egg’s blunt end using a thermal excitation of 40 °C.

**Figure 3:**
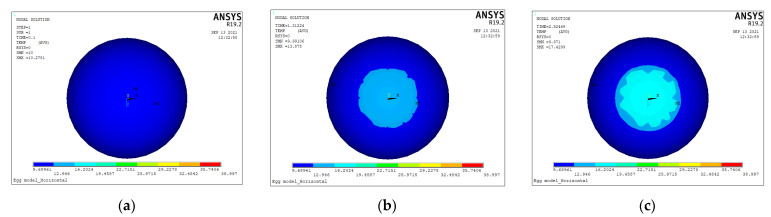

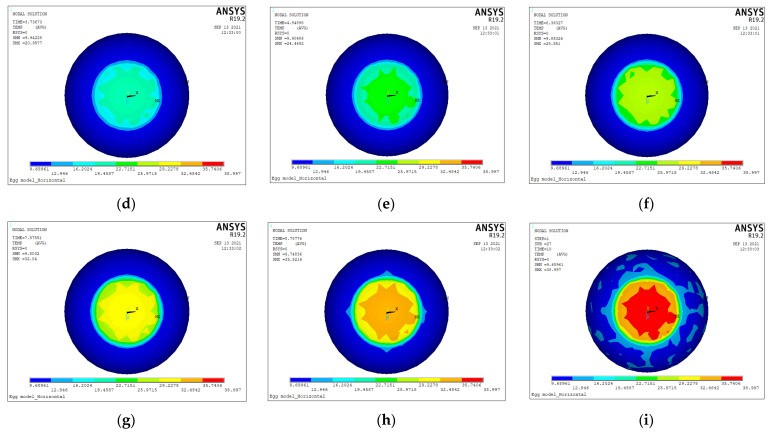
Dynamic cloud diagrams of H-EGG blunt end temperature over time. (**a**) *t* = 0.10000 s, (**b**) *t* = 1.31224 s, (**c**) *t* = 2.52449 s, (**d**) *t* = 3.73673 s, (**e**) *t* = 4.94898 s, (**f**) *t* = 6.36327 s, (**g**) *t* = 7.57551 s, (**h**) *t* = 8.78776 s, and (**i**) *t* = 10.00000 s.

The dynamic cloud plot of the temperature variation of the blunt end of the egg with time after being subjected to thermal excitation of 40 °C is shown in Figure 3. The egg air cell produced a temperature difference with the albumen at 1.31224 s, forming a clear boundary between them (Figure 3b). Starting at 6.36327 s, multiple temperature gradients accumulated at the junction of the egg air cell and the albumen as the heating time increased (Figure 3f). When the time reached 8.78776 s, the temperature of the albumen started to rise because it was subjected to both thermal convection and heat transfer from the eggshell (Figure 3h).

**Figure 4:**
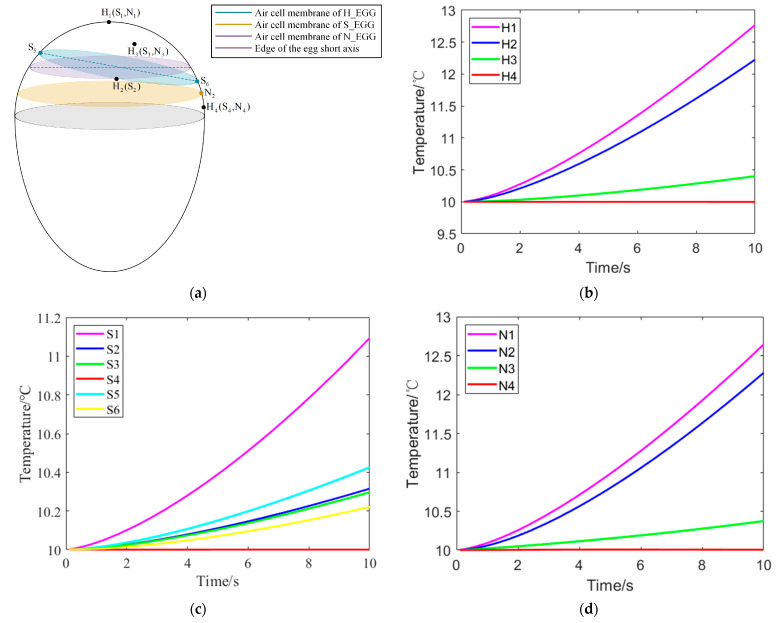
Schematic diagrams of the temperature variations of different nodes on eggs with time. (**a**) Location of nodes, (**b**) temperature profile of nodes in H-EGG model, (**c**) temperature profile of nodes in S-EGG model, and (**d**) temperature profile of nodes in N-EGG model.

To study the process of temperature change in different regions of the egg, the corresponding temperature change curves were drawn for nodes located at the blunt end of different egg models (Figure 4). In Figure 4a, H_1_(S_1_, N_1_) was located at the top of the egg’s blunt end, H_3_(S_3_, N_3_) was located inside the egg air cell, H_2_ was located on the edge of the air cell membrane in the H-EGG model, S_2_(S_5_, S_6_) was located on the edge of the air cell membrane in the S-EGG model, N_2_ was located on the edge of the air cell membrane in the N-EGG model, and H_4_ was located on the surface of the albumen.

The relationship between the temperature and distance of different nodes at the same time could be obtained from Figure 4. The distance between H_2_ and H_1_ in the H-EGG model was shorter than that between H_4_ and H_1_, and the temperatures of the three nodes from high to low were H_1_, H_2_, and H_4_ at the same time. Similarly, in the N-EGG model, the distance between N_2_ and N_1_ was shorter than that between N_4_ and N_1_, and the temperatures of the three nodes from high to low were N_1_, N_2_, and N4 at the same time. For the S-EGG model, the distances from each node to S_1_ were in the order of S_5_, S_2_, S_6_, and S_4_. At the same time, the temperatures of these nodes from high to low were S_5_, S_2_, S_6_, and S_4_. For the same egg model, the temperature at the top of the egg’s blunt end increased fastest, and the temperature growth rate at other positions on the eggshell surface was inversely correlated with the distance from the top of the egg’s blunt end. At the same time, the eggshell surface heated faster than the egg air cell, so the temperature of H_2_(S_2_, N_2_) was higher than that of H_3_(S_3_, N_3_).

At 2.119534 s, the temperature difference, Δ*T_H_*, between nodes in H-EGG model was:

(2) $$\Delta T_{H_{1}H_{2}}=0.07103\;{}^{\circ}C$$

(3) $$\Delta T_{H_{2}H_{4}}=0.22808\;{}^{\circ}C$$

At 1.750721 s, the temperature difference, Δ*T_S_*, between nodes in S-EGG model was:

(4) $$\Delta T_{S_{1}S_{2}}=0.06180\;{}^{\circ}C$$

(5) $$\Delta T_{S_{2}S_{4}}=0.07057\;{}^{\circ}C$$

At 1.963445 s, the temperature difference, Δ*T_N_*, between nodes in N-EGG model was:

(6) $$\Delta T_{N_{1}N_{2}}=0.07050\;{}^{\circ}C$$

(7) $$\Delta T_{N_{2}N_{4}}=0.17521\;{}^{\circ}C$$

When the eggs were subjected to thermal excitation from 1.31224 to 2.52449 s, there was a significant temperature difference between the egg air cell and albumen, so the thermal excitation time was set to 2 s. This could be achieved in the assembly line by matching the thermal excitation area with the assembly line speed.

### 2.2. Pulsed Thermographic Imaging System

#### 2.2.1. Sample Preparation

We purchased a total of 900 unfertilized, intact, and crack-free eggs from Panchu Mechanized Chicken Farm, Nanjing, Jiangsu Province, China; these eggs were produced within 24 h of the day of purchase. Among them were 450 pink-shelled eggs (mass 54.51 ± 9.37 g) and 450 brown-shelled eggs (mass 64.16 ± 7.94 g). The two types of eggs were divided into six groups: Pink-A (200 clean, pink-shelled eggs), Pink-B (200 dirty, pink-shelled eggs), Pink-C (50 clean, pink-shelled eggs), Brown-A (200 clean, brown-shelled eggs), Brown-B (200 dirty, brown-shelled eggs), and Brown-C (50 clean, brown-shelled eggs). After collecting thermal infrared images of Pink-A, Pink-B, Brown-A, and Brown-B on the first day, as well as measuring HU, all of these eggs were placed in a PQX-250L incubator (30 °C, RH 80%). Then, the same operation was performed every two days and the final measurements were obtained at 13 and 15 days.

#### 2.2.2. Egg Thermal Infrared Image Acquisition System

**Figure 5:**
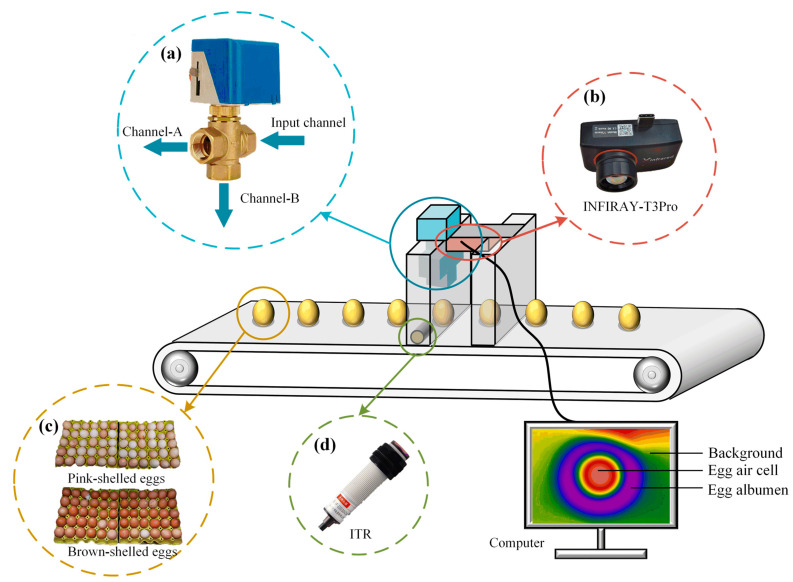
Schematic diagram of egg thermal infrared image acquisition device. (**a**) Solenoid valve, (**b**) thermal infrared imager, (**c**) egg samples, and (**d**) LED photoelectric switch.

A pulsed thermographic imaging system was built to facilitate data collection [34]. The eggs were placed on the conveyor belt and transported to the detection location (Figure 5). When an egg was sensed by a photoelectric switch, the solenoid valve was connected, and the egg was heated by hot air excitation from Channel-B. When the photoelectric switch could not sense an egg, the solenoid valve was disconnected, and the hot air excitation was released from Channel-A. The thermal infrared video of the egg-heating process was collected and saved by a computer-controlled infrared thermal imager. To ensure the stability of the eggs during the experiment, the running speed of the assembly line was set at 0.015 m/s, the time taken from the opening to closing of the solenoid valve was 3 s, and the time taken for the thermal excitation to be applied to the surface of the eggs was 2 s. A total of 1061 egg thermal infrared videos were collected from the thermal excitation process.

### 2.3. Modeling and Classification of Eggs Using Machine Learning

#### 2.3.1. Mathematical Mechanistic Analysis

ACH is an important indicator to determine the freshness level of eggs. We determined egg freshness by studying the correlation between the characteristics of the egg thermal infrared images and ACH.

**Figure 6:**
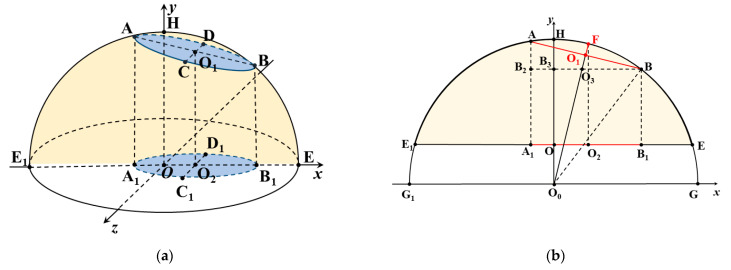
Schematic projection of the egg air cell. (**a**) Air cell projection of the egg’s blunt end and (**b**) cross-section of the egg’s blunt end on the xOy plane.

Because the egg’s blunt end is a spherical crown, the egg air cell membrane, which is a cross-section of the spherical crown, is circular and its projection on the egg equatorial plane is elliptical (Figure 6). When the egg air cell membrane is parallel to the egg equatorial plane, its projection is circular. In Figure 6a, the xOz plane is where the egg’s short axis lies, circle *O* is the egg’s short axis section, circle *O*_1_ is the egg air cell membrane, and ellipse *O*_2_ is the projection of circle *O*_1_ in the xOz plane.

Figure 6b is a section of the egg’s blunt end in the xOy plane shown in Figure 6a. The sector *EHE*_1_ is the egg’s blunt end profile, and *OE* is the radius of the short axis of the egg. The semicircular *GHG*_1_ is the semi-section of the egg’s blunt end (spherical crown) belonging to the sphere, and *O*_0_*G* is the radius of this sphere. *AB* is the diameter of the egg chamber membrane, and A1B1 is the projection of the egg chamber membrane diameter, that is, the short axis of the chamber projection. *O*_1_*F* is the egg air cell height, and ∠*ABB*_2_ is the angle between the air chamber membrane and the maximum radius section of the egg; the horizontal coordinates of *A* and *B* can be determined in the projection image as *x_A_* and *x_B_*, respectively.

Accordingly, it is known that: *O*_0_*H* = *O*_0_*G* = *O*_0_*B* = *R*, *O*_1_*A* = *O*_1_*B* = *R_a_*, *O*_2_*A*_1_ = *O*_2_*B*_1_ = *R_b_*, *O*_1_*F* = *h*, ∠*ABB*_2_ = α, and 0 < α < 90°.

(8) $$O_{1}O_{3}=\frac{O_{1}B}{\tan\theta }=\frac{R_{a}}{\tan\theta }$$

(9) $$O_{0}O_{3}=\frac{B_{3}O_{3}}{\sin\alpha }=\frac{BB_{3}-BO_{3}}{\sin\alpha }=\frac{x_{B}-R_{a}/\cos\alpha }{\sin\alpha }$$

(10) $$h=O_{0}F-O_{0}O_{1}=R-\sqrt{R^{2}-R_{a}{}^{2}}$$

It can be seen from this:

(11) $$O_{0}O_{1}=O_{0}O_{3}+O_{1}O_{3}=\frac{R_{a}R_{b}(R_{b}+x_{B})-R_{a}{}^{3}}{R_{b}\sqrt{R_{a}{}^{2}-R_{b}{}^{2}}}$$

(12) $$O_{0}O_{1}=\sqrt{O_{0}B^{2}-O_{1}B^{2}}=\sqrt{R^{2}-R_{a}{}^{2}}$$

Then, there is:

(13) $$h=\frac{R_{a}}{R_{b}\sqrt{R_{a}{}^{2}-R_{b}{}^{2}}}\left[\sqrt{\left(R_{a}{}^{2}-R_{b}{}^{2}\right)\left(R_{b}{}^{2}x_{B}^{2}+2R_{b}x_{B}+R_{a}{}^{2}\right)}-R_{a}R_{b}\left(R_{b}+x_{B}\right)+R_{a}{}^{3}\right]$$

In the thermal infrared image of an egg, ACH is related to the egg’s short axis length, the center of the short axis-located section, the long and short axes lengths of the projected ellipse, and the center of the projected ellipse, as expressed in Equation (13).

#### 2.3.2. Thermal Image Processing and Feature Selection

**Figure 7:**
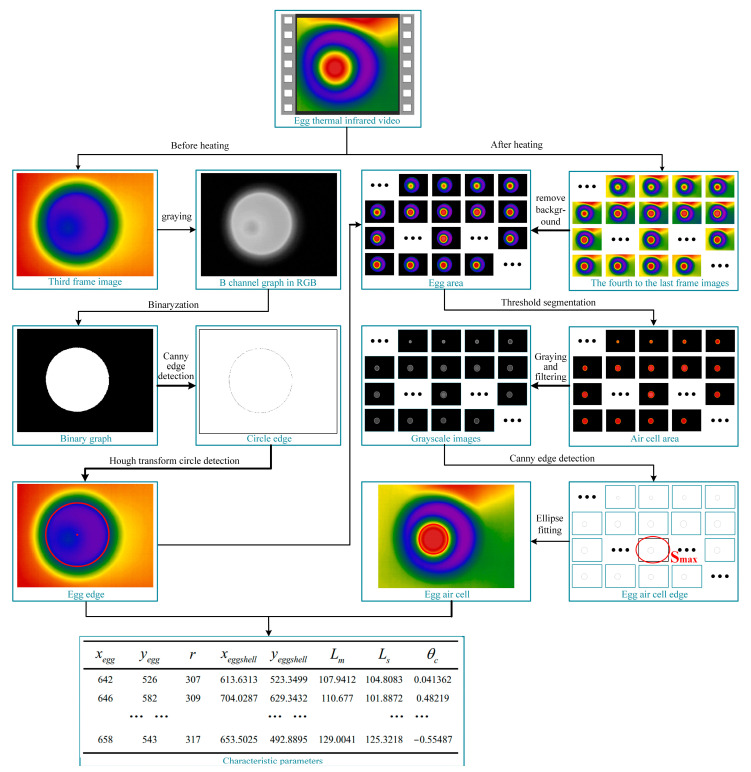
Feature extraction from the egg thermal infrared videos.

Frame selection, edge detection, feature extraction, statistical analysis, and feature selection were performed on 1061 thermal infrared videos of eggs using MATLAB software (Figure 7).

When the egg was not heated, its overall temperature remained the same, and there was only a temperature difference between the egg and the external environment. At this time, the inside of the egg was the lowest temperature in the whole image, i.e., the blue region of the thermal infrared image. The B-channel grayscale image in the RGB color space was selected to obtain the outside edge features of the egg. Its binary image was obtained using the maximum interclass variance method (Otsu), and the Canny edge operator [35] was used to extract the outside edge of the egg.

When the egg’s blunt end was thermally excited, there were three distinct temperature zones in the egg thermal infrared image: the egg air cell, the egg albumen, and the external environment. Among them, the temperature in the egg air cell was the highest, followed by the edge of the egg air cell, and the lowest temperature was in the egg albumen. Although the temperature of the external environment increased due to heat, there was a significant temperature difference with the egg albumen. Therefore, after the background removal operation of the egg thermal infrared video frame by frame, RGB threshold segmentation was used to extract the egg air cell area (red). The canny edge detection algorithm was used to detect the egg air cell edge, and the ellipse curve overlapping with the edge was fitted to obtain the edge information of the air chamber. Then, the egg air cell areas in all frames were compared, the edge information of the egg air cell was extracted from the frame, and the largest area was retained.

Finally, a total of 8 values, including the egg center coordinates (*x_egg_*, *y_egg_*), radius (*r*) of the egg circular edge, the center coordinates (*x_eggaircell_*, *y_eggaircell_*), long axis (*L_m_*), short axis (*L_s_*), and eccentricity angle (*θ_c_*) of the elliptical air cell were used as the characteristic parameters for egg freshness detection, allowing for the establishment of our egg freshness detection models. Based on the above features, the final extracted feature matrix was:

(14) $$\lambda_{1}=[x_{egg},y_{egg},r,x_{eggaircell},y_{eggaircell},L_{m},L_{s},\theta_{c}]$$

#### 2.3.3. Modeling and Classification

We established four egg freshness level discriminative models, namely the naive Bayes model (NBM), k-nearest neighbors (KNN), decision tree, and random forest (RF). NBM is an algorithm based on conditional independence assumption and Bayes’ theorem, which means that the freshness level of each egg sample was assumed to be independent of that of other eggs; it then calculated the conditional probabilities of the eggs belonging to each freshness level and divided them into the freshness level with the largest probability [36]. KNN classified the eggs by calculating the Euclidean distance between the training set and the samples to be tested in the feature space, taking the K samples with the closest distance; if most of these eggs belonged to a certain level of freshness, the eggs to be tested also belonged to this level [37]. The decision tree classification model reflected a mapping relationship between egg sample feature parameters and freshness, which was essentially a set of egg freshness detection rules generalized from a training set [28]. Random forest randomly extracted egg samples from the training dataset by the bootstrap method and generated different data subsets; it then established the decision tree model in turn to obtain a variety of egg freshness detection results, and finally, according to the voting results and the principle of majority voting, the egg freshness detection results of the random forest model were determined [38].

### 2.4. Modeling and Classification of Eggs Using Deep Learning

#### 2.4.1. Dataset

The original egg thermal infrared image dataset was derived from the thermal infrared videos of eggs taken on the assembly line, and 992 heated egg thermal infrared images were selected as the original sample data after screening. Then, the whole egg, the air cell area, and the background area were marked as “eggshell,” “egg air cell,” and “background,” respectively. The training sample dataset was completed by expanding the training images to 7470 through geometric transformations such as horizontal flip, vertical flip, and translation.

#### 2.4.2. Convolutional Neural Network

**Figure 8:**
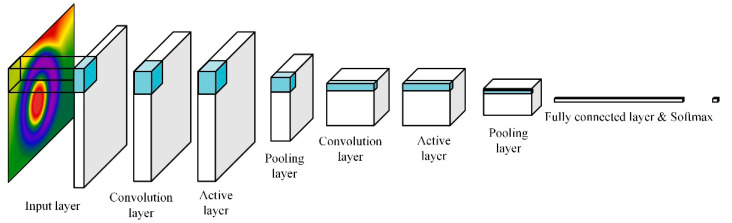
Schematic diagram of convolutional neural network structure.

A convolutional neural network (CNN) is a feed-forward neural network with convolution computation and deep structure, which generally includes an input layer, convolution layer, activation function, pooling layer, and full connection layer (Figure 8). It is widely used in image feature extraction and classification, image segmentation, and image restoration [39].

In the input layer, the egg thermal infrared images preprocessed by standardization, enhancement, scaling, and denoising were used as the input data. In the convolution layer, the feature of the input image data was extracted by sliding a fixed step through multiple different convolution kernels, and the feature map was output by a nonlinear activation function. The input images in this paper were three-channel RGB images, which needed convolution processing for each channel. The pooling layer was located in the middle of the continuous convolution layer and used a fixed-size pooling window to scan the feature map in order to obtain the maximum or average value in the window, thus creating a new image for feature reduction and information filtering. The fully connected layer was located behind the convolution and pooling layers, and its connection mode was to expand the output of the last convolution layer to the same level. In the output layer, the Softmax function was used to normalize the network. During the training process, the Dropout function was used to prevent neural network overfitting; that is, the Dropout function was set in the full connection layer, and 20% of the feature detectors were randomly ignored.

#### 2.4.3. Transfer Learning

Transfer learning applies the knowledge learned in the source domain to the target domain. In the case of less image data, transfer learning can update the bottom weights based on the weights trained by a large number of image datasets so as to reduce the amount of data required for model training and improve its efficiency [31]. VGG is the most commonly used model [40], and the image segmentation network used in this paper was based on it.

#### 2.4.4. VGGNet and SegNet Networks

**Figure 9:**
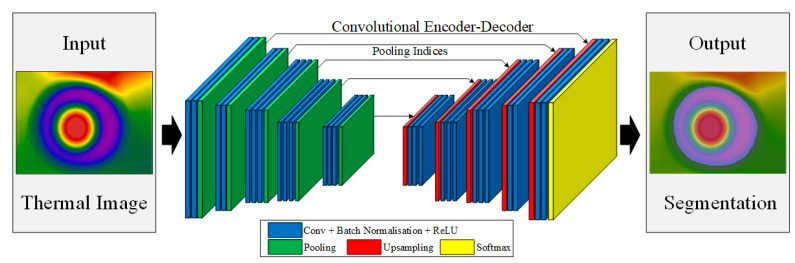
Schematic diagram of the SegNet network structure.

As seen in Figure 9, the left side of the SegNet network was the input egg thermal infrared image, the right side was the output of the image segmentation result, and the middle network structure was the whole process of semantic segmentation.

The SegNet egg thermal infrared image segmentation network consisted of two parts, an encoder and a decoder. The encoder network consisted of 13 layers of the convolution layer (the first 13 layers of VGG16) and 5 layers of the pooling layer. The VGG-16 convolutional neural network architecture was developed by Simonyan et al. [41]. The decoding network consisted of 5 layers of the upper sampling layer, 13 layers of the convolution layer, and one layer of the Softmax classification layer. The core of the SegNet network was the process of up-sampling using the maximum pooling location index [42]. After adding the final Softmax layer, the maximum probability of each pixel belonging to all categories was output through the feature extraction of the encoder and the spatial mapping of the decoder. Finally, the classification result map was output to complete the classification of egg thermal image pixel level.

#### 2.4.5. Neural Network Training and Image Segmentation

**Figure 10:**
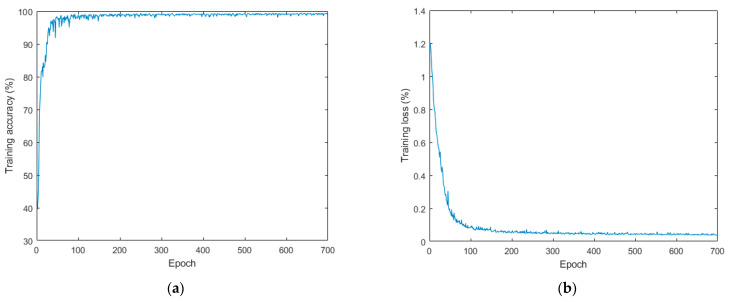
The training process of transfer learning. (**a**) The training set accuracy, and (**b**) the training set loss function.

The egg thermal infrared image dataset and pre-training weight parameters were prepared, and the dataset was randomly divided into training and validation sets at a ratio of 4:1. The dataset was run through the SegNet image segmentation neural network built by MATLAB for training. The model was trained with an initial learning rate of 0.03, a learning rate drop factor of 0.3, a learning rate drop period of 10 rounds, a maximum epoch of 30, and the data were shuffled in every epoch. In the training process, each weight parameter of the network model was updated iteratively. When the test accuracy of the validation set was no longer improved, the relatively optimal model parameters were saved. In a short period of time, the loss value of the training set decreased steadily and stabilized at 0.05, and the accuracy increased rapidly and fluctuated slightly between 0.97 and 1 (Figure 10).

**Figure 11:**
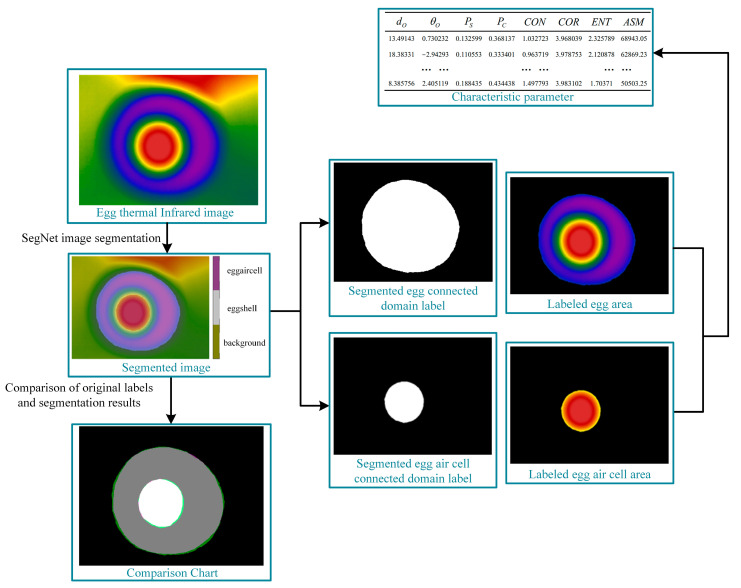
The connected domain of the whole egg and gas chamber after partition.

A testing set of 600 images was used to test the classification effect of the SegNet image segmentation model. Figure 11 shows that the egg air cell could be divided more accurately, and most of the egg albumen could be distinguished correctly; however, some areas were misclassified. Misclassification at the boundary between the egg and the background was common because the temperature of the eggshell increased upon heat excitation, resulting in the expansion of the thermal halo outside the eggshell. The results showed that the SegNet image segmentation model had a prominent segmentation effect on the egg thermal infrared images, with the overall accuracy reaching 98.25% (Table 2). The mIoU metric is a common performance metric used in deep learning algorithms based on region recognition and it was used to measure the performance of the SegNet network in thermal infrared image segmentation of the eggs. It was used to determine the degree of overlap between the detected regions of background, egg air cell, and albumen and the actual set of labels.

#### 2.4.6. Image Feature Extraction after SegNet Segmentation

The feature extraction process is shown in Figure 11. The 50 frames before and after the cessation of heating in the video were imported into the SegNet network model, and the image with the highest pixel value in the egg air cell area was selected because this image had the most accurate “egg air cell” information.

Then, according to the label information, the egg-connected and air cell-connected domains were obtained, and the centers of gravity of the two connected domains were obtained as (*x_egg_*, *y_egg_*) and (*x_eggaircell_*, *y_eggaircell_*), respectively; the area and perimeter were *S_egg_* and *C_egg_*, and *S_eggaircell_* and *C_eggaircell_*, respectively. The center of gravity distance (*d*) between the two connected regions, theta between the center of gravity line and horizontal line (*theta*), area ratio (*P_S_*), and perimeter ratio (*P_C_*) were obtained as follows:

(15) $$d=\sqrt{{\left(x_{egg}-x_{eggaircell}\right)}^{2}+{\left(y_{egg}-y_{eggaircell}\right)}^{2}}$$

(16) $$theta=arctan\frac{y_{egg}-y_{eggaircell}}{x_{egg}-x_{eggaircell}}$$

(17) $$P_{S}=\frac{S_{eggaircell}}{S_{egg}}$$

(18) $$P_{C}=\frac{C_{eggaircell}}{C_{egg}}$$

Meanwhile, the four texture features of contrast, correlation, entropy, and second-order moments of the egg-connected domain were extracted using the grayscale co-generation matrix (GLCM), where *G* (*i*, *j*) denotes the normalized grayscale co-generation matrix, and *i* and *j* are the matrix coordinates:

(19) $$CON=\sum_{i}\sum_{j}{\left(i-j\right)}^{2}G(i,j)$$

(20) $$COR=\frac{\sum_{i}\sum_{j}(ij)G(i,j)-\mu_{i}\mu_{j}}{S_{i}S_{j}}$$

(21) $$ENT=-\sum_{i}\sum_{j}G(i,j)\log G(i,j)$$

(22) $$ASM=\sum_{i}\sum_{j}{\left(G(i,j)\right)}^{2}$$

where

(23) $$\mu_{i}=\sum_{i}\sum_{j}i\cdot G(i,j)$$

(24) $$\mu_{j}=\sum_{i}\sum_{j}j\cdot G(i,j)$$

(25) $$S_{i}^{2}=\sum_{i}\sum_{j}G(i,j){\left(i-\mu_{i}\right)}^{2}$$

(26) $$S_{j}^{2}=\sum_{i}\sum_{j}G(i,j){\left(j-\mu_{j}\right)}^{2}$$

Based on the above features, the final extracted feature matrix was:

(27) $$\lambda_{2}=[d,theta,P_{S},P_{C},CON,COR,RNT,ASM]$$

#### 2.4.7. Support Vector Machine (SVM) Modeling and Classification

Based on the structural risk minimization (SRM) criterion, SVM maps the sample data from the low-dimensional space to the high-dimensional feature space through the nonlinear changes defined by the kernel function; thus, the linear inseparable data in the low-dimensional space have the possibility of linear separability in the high-dimensional space. The operation principle is to solve the optimal hyperplane with the maximum geometric interval for all vectors that can be properly partitioned into datasets [43].

## 3. Results and Discussion

### 3.1. Thermal Infrared Image of the Egg Blunt End

**Figure 12:**
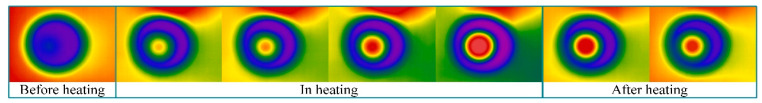
Image frames from the egg thermal infrared videos.

We captured thermal infrared video of the egg’s blunt end during the three states of no heating, heating, and cooling. Figure 12 shows some frames selected from an egg thermal infrared video. During the heating process of the egg’s blunt end, the egg air cell heated up rapidly and produced a large temperature gradient between it and the egg albumen. During the heat dissipation process, the temperature dropped faster in the area closer to egg albumen, and the temperature dropped the slowest at the top of the egg air cell. The experimental results were consistent with the finite element analysis results.

**Figure 13:**
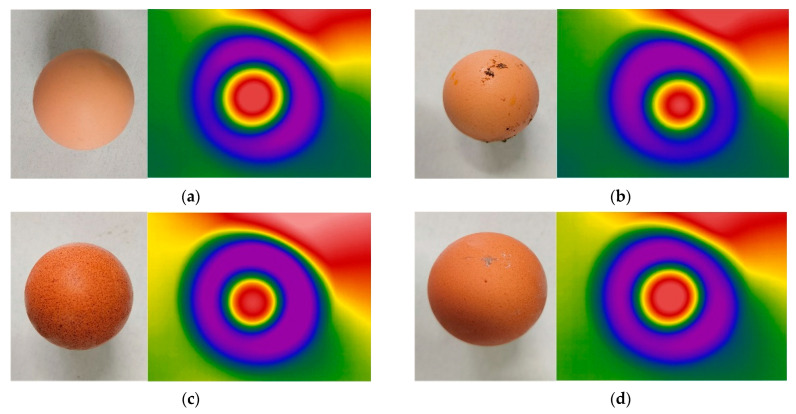
Thermal infrared images of different eggs. (**a**) Clean, pink-shelled egg, (**b**) dirty, pink-shelled egg, (**c**) clean, brown-shelled egg, and (**d**) dirty, brown-shelled egg.

We were able to obtain good thermal infrared images for eggs with pink clean shell, pink dirty shell, brown clean shell and brown dirty shell, respectively (Figure 13). Thus, shell color and cleanliness had no effect on the thermal infrared images of eggs.

### 3.2. Classification by Machine Learning

A total of 1061 egg thermal infrared videos were divided into training and validation sets in the ratio of 3:1, including 512 pink-shelled and 549 brown-shelled egg samples. The feature parameters of *x_egg_*, *y_egg_*, *r*, *x_eggaircell_*, *y_eggaircell_*, *L_m_*, *L_s_*, and *θ_c_* were extracted, and the labels were set according to HU. After normalization of the feature parameters, we used MATLAB to build four egg freshness detection models, including NBM, KNN, decision tree, and RF. The value of k within the KNN model was determined to be 3 after 5-fold cross-validation. When building the RF model, the initial number of leaf nodes was set from 5 to 500, and it was found that 5 leaf nodes and 100 trees were more appropriate after running the model. Among them, the RF egg freshness detection model achieved the best accuracy—94.09% in the training set and 92.32% in the validation set (Table 3). Therefore, the RF model was finally selected for the detection of egg freshness.

The testing set was used to test the classification effect of the RF egg freshness detection model. A total of 146 egg thermal infrared videos were tested in the testing set, of which 62 were grade AA, 54 were grade A, 19 were grade B, and 11 were grade C. The extracted feature values were the input for the RF egg freshness detection model for freshness grading, and the detection accuracy was 91.78% (Table 4).

### 3.3. Classification by SVM with Deep Learning

From the training and validation sets, we extracted the characteristic parameters, established the characteristic matrix, and set the egg level labels according to HU. To deal with the egg freshness multi-classification problem, SVM was set up as C-SVC, and the results of SVM egg freshness detection models under different kernel functions were investigated (Table 5).

Through practical experimental comparisons, the best detection results were obtained using the RBF kernel as the kernel function of the SVM, and the accuracies of the training and validation sets were 98.97% and 95.14%, respectively; the penalty coefficient was determined to be 111.4305, and the gamma value was 0.5743 by the cross-validation method. Then, the testing set was used to test the classification effect of the SVM egg freshness detection model. The test set comprised a total of 146 egg thermal infrared images, of which 62 were class AA, 54 were class A, 19 were class B, and 11 were class C. The extracted features matrix was the input for the SVM egg freshness detection model, and the detection accuracy reached more than 94% (Table 6).

### 3.4. Results Analysis

The experimental results showed that only the temperature values of the blunt ends of eggs with different freshness levels, air cell tilt angles, and shell states were different under thermal excitation, and the overall temperature change pattern was the same. Moreover, when the egg’s blunt end was heated at 40 °C for 2 s, a large temperature difference between the egg air cell and egg albumen was observed, with a clear boundary in the thermal infrared images.

Compared with the four egg freshness detection models of NBM, KNN, decision tree and RF, the deep neural network was able to extract richer data features from the egg thermal infrared images. The SVM egg freshness detection model built in combination with the SegNet neural network model had higher detection accuracy, with 94.52% detection accuracy.

We also found that the accuracy of feature parameter extraction from the egg thermal infrared images was reduced due to the tilt of the egg air cell, which affected the accuracy of the egg freshness detection model. Moreover, since there was a time difference between heating and detection, the thermal shock needed to be set at a specific distance before detection. A cooling environment could be added before thermal excitation to increase the temperature difference between the eggs and the environment, thus avoiding a detection problem when the eggs warm up too quickly at room temperature.

## 4. Conclusions

This study established the possibility of using infrared thermography to detect the freshness of different varieties of eggs with different levels of soiling. In our study, three thermal finite element analysis models of eggs with different freshness levels were developed using ANSYS (Figure 1). The most prominent boundary between the two regions of the egg and the gas chamber was determined to exist when the egg was subjected to thermal excitation treatment at 40 °C for 2 s (Figure 4). This provided a theoretical basis for an egg freshness detection method based on infrared thermography (Figure 3).

In this study, we determined the freshness of eggs based on the height of the gas chamber, so we used a mathematical model to establish the correlation between the height of the egg gas chamber and the size of the egg air cell membrane. Two image segmentation methods based on color features and the SegNet neural network, respectively, were used for image processing of thermal infrared images of eggs. The results of both methods were similar when segmenting only horizontally tilted thermal infrared images of fresh eggs, but SegNet gave more accurate semantic segmentation results for the eggs with different freshness levels and shell colors studied in this experiment. The segmentation method based on color features took the coordinate positions in the mathematical model and extracted the air chamber coordinates as the feature parameters to establish the feature matrix (λ_1_), while the segmentation method based on the SegNet neural network used the image center of gravity and grayscale co-generation matrix as the feature parameters (λ_2_). The detection accuracy of the established egg freshness detection models were above 90%, effectively determining the freshness of eggs. This method could be used to test the freshness of eggs on an automated line.

At the same time, we impose a restriction on the method, requiring that the thermal shock be set at a certain distance before detection. As the image edges in thermal infrared imaging are affected by temperature gradients, a halo will appear, which is related to the temperature difference between the egg and the environment (Figure 12); the larger the temperature difference, the smaller the halo. The eggs can be cooled before thermal excitation to increase the temperature difference, reduce the generation of halos, and improve the detection accuracy. The egg freshness detection model can also be optimized and improved to achieve faster results. We will further investigate the application of infrared thermography in fertilized egg detection to improve embryo hatching rates.

## Figures and Tables

**Table 1:** Thermo-physical properties of the egg model.

	Thermal Conductivity (W/mK)	Specific Heat (J/kg K)	Density (kg/m^3^)
Shell	0.4560	888.0	2300.000
Air cell	0.0239	1008.5	1.265
Albumen	0.5900	3560.0	1035.000

**Table 2:** Results of SegNet egg thermal infrared image segmentation.

	All Accuracy			Boundary Fitting Score		Intersection over Union	
	Accuracy	Global Accuracy	Mean Accuracy	BF Score	Mean BF Score	IoU	mIoU
Background	0.9862	0.9825	0.9834	0.9868	0.9822	0.9835	0.9626
Albumen	0.9793			0.9783		0.9502	
Air cell	0.9901			0.9924		0.9527	

**Table 3:** Comparison of training effects of different classification models.

Egg Freshness Detection Model	Training Set Accuracy (%)	Validation Set Accuracy (%)
NBM	86.91	86.03
KNN	91.06	87.16
Decision Tree	84.96	81.82
RF	94.09	92.32

**Table 4:** Classification results of egg freshness in the testing set by the RF egg freshness detection model.

Egg Freshness Grade	Sample Size	Correct Discriminant	Accuracy (%)
AA	62	58	91.78
A	54	51	
B	19	16	
C	11	9	

**Table 5:** Comparison of training effects of different kernel functions of SVM egg freshness detection models.

Kernel Function Type	Training Set Accuracy (%)	Verification Set Accuracy (%)
Linear	90.53	90.92
Polynomial	72.32	72.03
Radial Basis Function (RBF)	98.97	95.14

**Table 6:** Classification results of egg freshness in the testing set by the SVM egg freshness detection model.

Egg Freshness Grade	Sample Size	Correct Discriminant	Accuracy (%)
AA	62	60	94.52
A	54	51	
B	18	16	
C	12	11	
